# Statewide agent-based model for management of chronic wasting disease in white-tailed deer: *PAOvCWD*

**DOI:** 10.1016/j.mex.2026.103823

**Published:** 2026-02-17

**Authors:** Nathaniel H. Wehr, Christopher S. Rosenberry, David Stainbrook, Maureen Staats, Andrea L. Korman, W. David Walter

**Affiliations:** a Pennsylvania Cooperative Fish and Wildlife Research Unit, The Pennsylvania State University, 419 Forest Resources Building, University Park, PA 16802, USA; b Bureau of Wildlife Management, Pennsylvania Game Commission, 2001 Elmerton Ave., Harrisburg, PA 17110, USA; c U.S. Geological Survey, Pennsylvania Cooperative Fish and Wildlife Research Unit, The Pennsylvania State University, 419 Forest Resources Building, University Park, PA 16802, USA

**Keywords:** Individual-based model, *Odocoileus virginianus*, Simulation, Transmission, ABM, CWD, IBM

## Abstract

Chronic wasting disease (CWD) is an always-fatal disease infecting wild cervids globally. Ecologically and economically important, CWD presents a challenge for managing white-tailed deer (*Odocoileus virginianus*). We built an agent-based model to simulate CWD transmission and assess potential management actions that could slow disease spread: *PAOvCWD*. We developed *PAOvCWD* using contact rates and other behavioral and ecological metrics estimated from deer monitored in Pennsylvania, USA. We programmed potential management responses (e.g., culling, altered hunter harvest) for all 22 Pennsylvania wildlife management units and validated the efficacy of *PAOvCWD* using a deer population in south-central Pennsylvania infected with CWD for > 10 years. To support applications of our model, we developed a user-friendly R pipeline that allows implementation with relatively minor modifications. Our pipeline includes four steps:

• Steps 1 and 2 generate regional percent forest cover rasters and curate population-level demographics.
• Step 3 initializes landscapes and agents using our *PAOvPOP* model.
• Step 4 assesses CWD transmission and management responses using our *PAOvCWD* model.

## Graphical abstract

**Figure:**
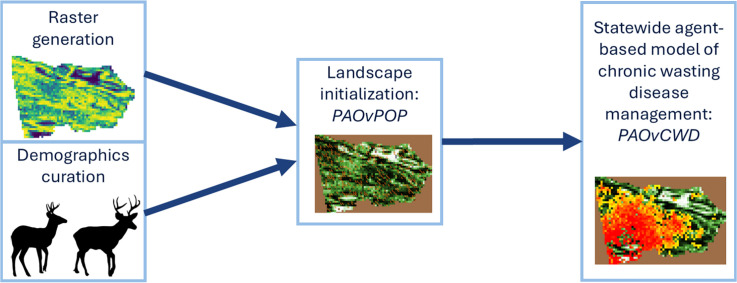

**Table:** Specifications table.

**Subject area**	Agricultural and Biological Sciences
**More specific subject area**	Wildlife Ecology and Management
**Name of your method**	*PAOvCWD*
**Name and reference of original method**	A. Belsare, M. Gompper, B. Keller, J. Sumners, L. Hansen, and J. Millspaugh, Size matters: sample size assessments for chronic wasting disease surveillance using an agent-based modeling framework, MethodsX 7 (2020) 100953.
**Resource availability**	All code needed to replicate this method was published as N.H. Wehr, C.S. Rosenberry, D. Stainbrook, M. Staats, A.L. Korman, W.D. Walter, Agent-based modeling of chronic wasting disease transmission among white-tailed deer and potential management responses, Software Release, U.S. Geological Survey, Reston, VA, USA (2025), https://doi.org/10.5066/P1CTCVRS.

## Background

Chronic wasting disease (CWD) is an always-fatal transmissible spongiform encephalopathy that infects wild and domestic cervids (family: Cervidae) [1]. CWD has been detected among cervids in Canada, Finland, Norway, Sweden, South Korea, and the United States [2]. In the United States, state-level monitoring and management responses cost, on average, $500 thousand annually per state [3,4]. White-tailed deer (*Odocoileus virginianus*) are abundant in the eastern United States where they are an economically valuable contributor to wildlife viewing and hunting [5,6]. White-tailed deer populations are heavily influenced by CWD, warranting adequate modeling of transmission in support of white-tailed deer management [[3], [7]].

The *MOOvPOPsurveillance* and *MIOvCWDdy* agent-based models (ABMs) were developed to support CWD management in Missouri and Michigan, USA [[8], [9], [10], [11], [12]]. These models were programmed in NetLogo [13] with the primary goal of determining monitoring strategies needed for estimating CWD prevalence and the influence of hunter harvest on CWD transmission [11,12]. These models have since been applied in Indiana, USA as *INOvCWD* [14], adapted to assess the influence of hemorrhagic disease on CWD transmission [15], and adapted to assess CWD transmission dynamics among reindeer (*Rangifer tarandus*) [16]. Other ABMs assessing CWD transmission among white-tailed deer [17,18] and mule deer (*Odocoileus hemionus*) [19] used the Repast Symphony [20] or Python (Python Software Foundation, Wilmington, Delaware, USA) platforms in lieu of NetLogo.

Limitations of some of the above approaches include a lack of locally sourced movement metrics for white-tailed deer, the use of artificially generated as opposed to real landscapes, and difficulty replicating or modifying the protocols. In this manuscript, we describe the computational methodology we developed to overcome these challenges. Our methodology includes four modifiable R [21] scripts for developing model inputs and running simulations. These scripts are intended for analytical assessments of CWD management in Pennsylvania as well as easier modification and application of ABMs to new regions. Our ABM implements considerably modified versions of the *INOvPOP* [22] and *INOvCWD* [14] models. Our *PAOvPOP* and *PAOvCWD* models offer additional options for management responses and incorporate real-world information on deer demographics, movements, and landscapes covering the entirety of Pennsylvania, USA. In summary, the methodology outlined below is intended for easier assessment of management responses to CWD including adaptation of these methods to other regions and species.

## Method details

We developed an R pipeline that includes four steps allowing users to replicate our analyses or modify them in support of their own unique applications. All steps are completed in R [21], but Steps 3 and 4 are reliant on NetLogo [13] for programming and processing the ABMs. Step 1 generates landscape rasters to be imported into NetLogo. Step 2 curates demographic information for the species of interest (i.e., white-tailed deer; hereafter, deer). Step 3 imports landscape rasters and demographic information and establishes the initial ABM landscape using our *PAOvPOP* model. Step 4 simulates CWD transmission and the influence of management responses using our *PAOvCWD* model. This manuscript is a guide for application and reference for modification of our R pipeline and NetLogo models using the code in Wehr et al. [23]. We do not provide recommendations for analyzing resultant outputs, as analyses should be specifically tailored to individuals’ unique research questions.

### Step 1: generating rasters

The goal of Step 1 is to generate rasterized landscapes of percent forest cover that will be used in the *PAOvPOP* and *PAOvCWD* models in Steps 3 and 4. NetLogo requires rasters to be imported as American standard code for information interchange (ascii) files following a specific format, and each study area requires its own unique raster ascii file. Our R pipeline uses publicly available shapefiles and land cover data to generate, modify, and export the appropriate files. Our approach relies on the *dplyr* [24], *FedData* [25], *sf* [26,27], *readr* [28], and *terra* [29] R packages.

Our study areas included all the wildlife management units (WMUs) in Pennsylvania, USA. We downloaded a single shapefile containing borders for all the Pennsylvania WMUs from a public repository [30]. This shapefile was structured as 23 distinct polygons; to improve processing efficiency for importing land cover data, we generated an additional shapefile wherein the boundaries between each WMU were dissolved (i.e., an outline of Pennsylvania). Corresponding to their regions of interest, users can modify these steps by importing and dissolving their own study area shapefiles as appropriate.

We used the 2019 National Land Cover Database (NLCD) [31] to estimate percent forest cover for each raster cell in each WMU. We imported the NLCD raster at 30-m resolution covering our entire Pennsylvania shapefile. The NLCD categorizes land cover types into 20 unique classifications assigned numeric identifiers [31]. Based on results from previous CWD modeling efforts [32], we combined land cover into two categories likely influencing deer movements and CWD spread in Pennsylvania. We reclassified 30-m cells assigned shrub, woody wetland, and deciduous, evergreen, or mixed forest designations as “forested” (value = 1) and 30-m cells assigned open water, herbaceous wetland, developed (all classifications), barren, grassland, pasture, and crops designations as “unforested” (value = 0), reflecting habitats with available cover. We aggregated 30-m cells by a factor of 53.6 to produce raster cells approximately 1 mile^2^ (2.59 km^2^). The resultant output raster was composed of 1 mile^2^ (2.59 km^2^) cells each containing a value equivalent to percent forest cover as calculated by the mean of all forested (value = 1) and unforested (value = 0) 30-m cells composing the original NLCD raster for the entirety of Pennsylvania. We used 1 mile^2^ cells in lieu of 1 km^2^ cells because land managers in the United States typically operate at this scale [9,11]. The importation and adaptation of NLCD rasters requires minimal modifications (e.g., R environment object names) for application to other study areas.

Using the statewide percent forest cover raster, we generated 22 unique rasters representing each current Pennsylvania WMU—WMU 2H became part of WMU 2G in 2023 after the public repository was last updated. We subdivided our initial shapefile containing all 23 WMUs into 23 unique shapefiles. We merged (i.e., unioned) WMUs 2H and 2G and removed WMU 2H corresponding to recent boundary modifications. For each of the 22 current WMUs, we cropped the statewide 1 mile^2^ (2.59 km^2^) percent forest cover raster and masked percent forest cover values occurring outside each WMU using an iterative loop.

**Fig. 1:**
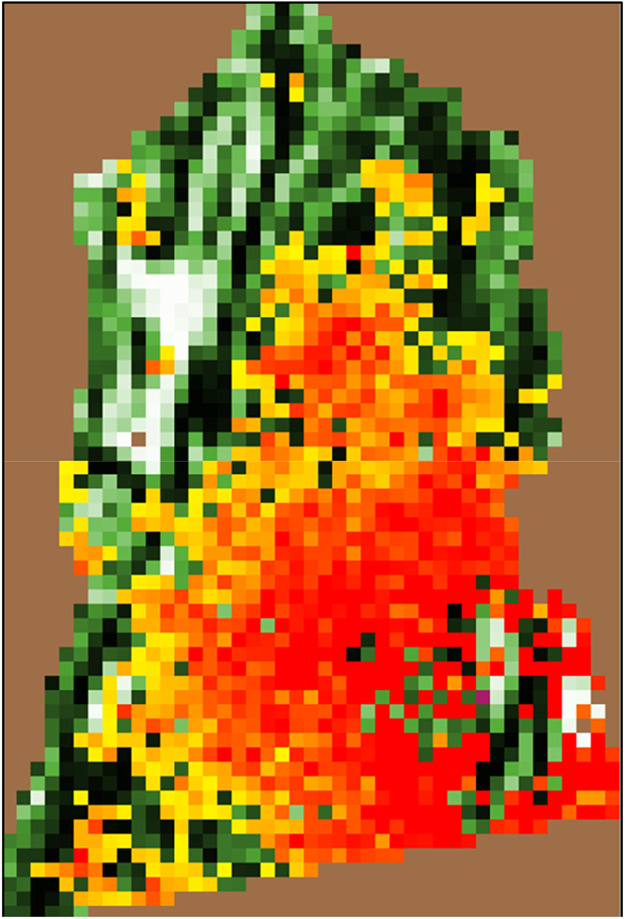
Wildlife management unit (WMU) 4A of Pennsylvania, USA as depicted in the *PAOvCWD* interface. Percent forest cover is represented by green shading scaled from light to dark (low to high values). Brown patches represent those deemed unsuitable for deer (i.e., < 5 % forest cover) or outside the WMU. Percent forest cover was derived from the 2019 National Land Cover Database [31]. Yellow to red gradient patches correspond to the proportion of deer infected with chronic wasting disease.

To achieve proper formatting for ascii files, we exported rasters as ascii files wherein “no data” values equaled 0. We then imported these ascii files, modified the no data value to equal -9999, and exported the modified ascii files directly to the appropriate folder within the NetLogo model structure thereby concluding the first step in our R pipeline. This process was necessary because NetLogo interprets ascii file values differently than R and ArcGIS Pro (Esri, Redlands, California, USA), and users should expect to complete this modification when generating their raster ascii files. In the modified ascii files, raster cells outside the WMU boundaries had a value of 0, and raster cells inside the boundary had values between 0 and 1. The no data value was set to -9999, which resulted in rectangular raster ascii files that could be interpreted by NetLogo. In the *PAOvPOP* and *PAOvCWD* models, raster cells (i.e., patches) with percent forest cover < 0.05 are deemed unsuitable deer habitat [9], which results in a functional border that matches the user’s study area border (Fig. 1); more details are provided in the appropriate sections below.

### Step 2: curating demographics

The goal of Step 2 is to generate a comma-separated values (csv) file containing demographic information for deer corresponding to each study area ascii generated in Step 1. This csv file is imported into NetLogo in Step 3 to provide detailed information for each study area’s initial populations. This step relies on data curation using the *dplyr* package [24]. Users may optionally generate the necessary csv file in Excel (Microsoft, Redmond, Washington, USA) or their preferred software. In support of independent user modification, Table 1 depicts the contents of the csv file produced by our R pipeline; columns are defined in the following paragraph.

**Table 1:** Demographic summaries for white-tailed deer (*Odocoileus virginianus*) specific to each wildlife management unit in Pennsylvania, USA. Values represent the 12-year average inclusive of 2012–2023 estimated using sex-age-kill models for white-tailed deer in Pennsylvania [33,34]. Column headings reflect thoseused in the comma-separated values file imported to *PAOvPOP* and *PAOvCWD*.

Unit	Population_Density	Sex_ratio	Prop_A_Females	Prop_Y_Females	Prop_A_Males	Prop_Y_Males	Adult_Male_Harvest	Yearling_Male_Harvest	Antlerless_Harvest_Rates
1A	33	217	0.51	0.23	0.20	0.38	0.60	0.28	0.20
1B	33	204	0.55	0.24	0.25	0.39	0.57	0.26	0.18
2A	31	189	0.55	0.25	0.29	0.39	0.55	0.24	0.21
2B	30	194	0.51	0.25	0.23	0.39	0.58	0.27	0.31
2C	27	231	0.53	0.24	0.17	0.43	0.64	0.31	0.16
2D	45	209	0.55	0.23	0.24	0.39	0.57	0.26	0.18
2E	38	217	0.55	0.24	0.22	0.41	0.59	0.28	0.16
2F	35	233	0.61	0.20	0.28	0.36	0.54	0.24	0.11
2G	20	213	0.65	0.18	0.38	0.31	0.46	0.18	0.10
3A	33	221	0.63	0.19	0.34	0.33	0.50	0.21	0.12
3B	30	201	0.58	0.21	0.31	0.35	0.51	0.22	0.14
3C	33	198	0.60	0.21	0.33	0.35	0.50	0.21	0.17
3D	18	167	0.59	0.24	0.39	0.36	0.48	0.20	0.17
4A	21	205	0.57	0.25	0.26	0.44	0.57	0.26	0.23
4B	30	231	0.58	0.22	0.23	0.40	0.58	0.27	0.17
4C	33	209	0.55	0.23	0.24	0.39	0.57	0.26	0.13
4D	26	204	0.59	0.23	0.29	0.39	0.53	0.23	0.14
4E	36	214	0.55	0.24	0.22	0.42	0.59	0.28	0.15
5A	21	233	0.58	0.23	0.23	0.41	0.58	0.27	0.20
5B	29	212	0.52	0.25	0.19	0.42	0.61	0.30	0.18
5C	32	195	0.52	0.23	0.24	0.37	0.56	0.25	0.24
5D	16	182	0.54	0.21	0.32	0.31	0.49	0.20	0.30
**Statewide**	**30**	**204**	**0.56**	**0.23**	**0.26**	**0.38**	**0.55**	**0.25**	**0.17**

We estimated demographic parameters for each study area using 12-year averages from the Pennsylvania sex-age-kill model during 2012–2023 [33,34]. Summary data used in these calculations was provided by the Pennsylvania Game Commission (PGC; [23]). Using the summary data, we generated a dataframe including 10 columns: *Unit* (i.e., study area), *Population_Density* (deer / mile^2^), *Sex_Ratio* (number of females per 100 males), *Prop_A_Females* (proportion of females ≥ 30 months of age), *Prop_Y_Females* (proportion of females approximately 18 months of age), *Prop_A_Males* (proportion of males ≥ 30 months of age), *Prop_Y_Males* (proportion of males approximately 18 months of age), *Adult_Male_Harvest* (adult male harvest rate), *Yearling_Male_Harvest* (juvenile male harvest rate), and *Antlerless_Harvest_Rates* (harvest rate for females of all ages and males < 12 months of age). We did not calculate proportions of females and males approximately 6 months of age as the *PAOvPOP* model structure accounts for these missing proportions. We additionally calculated a statewide average by taking the mean of the 22 WMUs for each column (Table 1).

### Step 3: initializing populations

The goal of Step 3 is to generate the initial population of deer (i.e., agents) that will be used in Step 4 to simulate CWD transmission. The population initialization process uses the *PAOvPOP* model, which is described below. Step 3 uses the *nlrx* package to run NetLogo in the background without opening the software [35]. Users must have NetLogo and Java (Oracle, Austin, Texas, USA) installed to implement *nlrx*. Additionally, users must copy the *csv* and *gis* NetLogo extensions from the NetLogo extensions folder to the same folder as the *PAOvPOP* model to use our R pipeline—users may alternatively copy the *PAOvPOP* model to the NetLogo extensions folder, but we viewed this as the less intuitive file structure. *PAOvPOP* is programmed to export the necessary csv files containing initialized populations. As such, running *PAOvPOP* using either the NetLogo interface or Step 3 of our R pipeline will produce the csv files necessary for Step 4.

Our implementation of the *nlrx* package followed Salecker [36]’s vignette with appropriate modifications. Corresponding to *nlrx* package requirements, we defined internal file paths to our Java and NetLogo installations as well as the *PAOvPOP* model. We then initialized our experimental matrix following *nlrx* formatting. Parameters important for the user to define in the *nlrx* format for *PAOvPOP* include *runtime* (i.e., the number of months the model should be run for), *region* (i.e., the name of the study area), *recommended_parameter_values* (i.e., application of Pennsylvania statewide average harvest mortality regardless of region), and *output* (the post-harvest population csv file is required for Step 4). We set run time to 25 months to represent two years of natural population fluctuation plus an additional month to activate the appropriate *PAOvPOP* action for exporting the resultant csv files. We used the *PAOvPOP* recommended parameter values for all WMUs. Finally, we initialized a distinct *PAOvPOP* simulation for each WMU and exported the post-harvest population csv.

We used the *future* package [37] to assign each simulation to a separate computing core. The *nlrx* package was designed to incorporate *future* package options, and the *split* parameter within the *run_nl_all* function may be used to establish the number of cores dedicated to simulations [35]. A caveat to this approach is that the number of simulations must be divisible by the number of assigned cores. In our case, we simulated 22 WMUs using 11 cores, which typically required approximately three hours of computational wall time using an i7 processor (Intel, Santa Clara, California, USA) with 64 GB of random access memory (RAM).

### Step 4: simulating CWD management

The goal of Step 4 is to simulate CWD transmission and the influence of potential management responses on disease spread. Step 4 is similar to Step 3 because it is another application of the *nlrx* package [35] to operate NetLogo models; in this case, we used the *PAOvCWD* model, which is described below. As with *PAOvPOP*, users must copy the appropriate NetLogo extensions (i.e., *csv* and *palette*) from the NetLogo extensions folder to the same folder as the *PAOvCWD* model to use our R pipeline. Due to the computationally intensive nature of the *PAOvCWD* model, we produced two separate options in our R pipeline. The first is an R markdown file similar in structure and content to that implemented in Step 3. In this R markdown, we define file paths to Java, NetLogo, and *PAOvCWD*, develop an *nlrx*-formatted model matrix, and use the *future* package to define the number of computational cores being used for simulations. Using this format, individual simulations of WMUs typically required 4–8 h of computational wall time using an i7 processor with 64 GB of RAM. Our objective was to assess several well-replicated management scenarios covering large study areas; as such, the second set of code is an R script and accompanying text file, which we used to implement simulations on a high-performance computing (HPC) cluster with 8 GB of RAM per core.

Whether implementing Step 4 on a personal computer or HPC cluster, the user must define their model matrix formatted for *nlrx*. We set run time equal to 121 months representing 10 years plus a single month to activate the appropriate *PAOvCWD* action for exporting results. We defined the following parameters for each simulation. The study area (WMU) being simulated (*cwd_region*) a Boolean true or false parameter (*statewide_average*) that was always true in our validation models indicating statewide average harvest rates were used rather than WMU-specific harvest rates. Implementing the statewide average was necessary to maintain model stability because population estimates were strongly influenced by female harvest rates. We also defined a Boolean true or false parameter (*analysis*) necessarily set to true when using the *nlrx* package—the false option is for visual simulations within the NetLogo interface. The number of deer initially infected with CWD (*infection_seed*) was always set to 3 as values < 3 sometimes resulted in CWD being eliminated from the landscape prior to management actions; 3 was also the number of deer initially identified as being infected with CWD in our validation data [38,39]. Six parameters defined the percentage of each demographic class being tested for CWD from among hunter-harvested deer; these percentages were kept equal across demographic classes but were varied to influence time-to-detection. Options for management responses (*response*) included “None”, “Targeted_Culling”, “Add_DMAP”, “Alter_Antlerless_Harvest”, “Alter_Antlered_Harvest”, and “All” (Table 2). Depending on the intended management strategy we modified response parameters for percentage of post-harvest deer to be culled within the defined culling area (*targeted-culling-percentage*), size of culling target area (*culling_area*), frequency of culling efforts (*effort*) as none, once, or repeated, increase in harvest rate within the defined deer management assistance program (DMAP) area (*added-dmap-harvest*), size of implemented DMAP (*dmap_area*), change in antlerless harvest rate (*added-antlerless-harvest*), change in yearling male harvest rate (*added-yearling-harvest*), and change in adult male harvest rate (*added-adult-harvest*). We also defined export parameters such that we recorded the total count of deer, proportion of deer infected with CWD, whether or not CWD had been detected among hunter-harvested deer, number of 1 mile^2^ (2.59 km^2^) cells in which ≥ 1 deer was infected with CWD, and distance from the initially infected cell to the furthest infected cell at each timestep (i.e., month).

**Table 2:** Management responses that can be implemented in response to detection of chronic wasting disease (CWD) in the *PAOvCWD* model.

Management response	Description
None	No management response will occur. If a user inputs values associated with other management responses, these values will be ignored.
Targeted culling	Upon detection of CWD, the location of each positive test from that year is recorded. In December of the following year, deer will be culled immediately after the hunter harvest mechanism is completed. The number of deer culled is defined by a user-selected percentage (e.g., 50 % of deer in the target area). The size of the area (None, 1 mile^2^, 5 miles^2^, 9 miles^2^, or 25 miles^2^; 2.59 km^2^, 12.95 km^2^, 23.31 km^2^, or 64.75 km^2^) is also defined by the user and centered on the location of the previous year’s positive test(s). An effort parameter decides whether no culling will occur (None), culling will occur only following the first year of detection (Once), or culling will occur each year corresponding to available detections (Repeated). All culled deer are monitored for CWD and can inform the subsequent year’s management response.
Deer Management Assistance Program (DMAP)	Upon detection of CWD, the location of each positive test from that year is recorded. A DMAP subunit is then generated using these locations. Each year following the creation of the DMAP subunit(s), antlerless harvest within the designated area is increased by a user-defined input (e.g., user input = 10 %; antlerless harvest is increased from 17 % to 27 % within the DMAP subunit). The size of the DMAP subunits is also user-defined (None, 9 miles^2^, 25 miles^2^, 49 miles^2^, 81 miles^2^; 23.31 km^2^, 64.75 km^2^, 126.91 km^2^, or 209.79 km^2^) and centered on the location(s) of the initial positive detection(s). Overlapping DMAP subunits are merged into a single larger subunit.
Alter antlerless harvest	Following detection of CWD, the antlerless harvest value for the entire study area will be altered following a user input in all subsequent years of the simulation (e.g., user input = 8 %; antlerless harvest is increased from 17 % to 25 % for all remaining years following CWD detection).
Alter antlered harvest	Following detection of CWD, the yearling male and adult male harvest values for the entire study area will be altered in all subsequent years of the simulation following user inputs (e.g., user input = 25 % for yearling males and -5 % for adult males; yearling male harvest is increased from 25 % to 50 %, and adult male harvest is decreased from 55 % to 50 %).
All	If the “All” management response is selected, all of the above management responses (excepting “None”) will be implemented following their respective mechanisms. If, for example, a user wishes to implement both targeted culling and antlerless harvest, they would set the management response option to “All”, their user inputs for culling and antlerless harvest as intended, and their DMAP and antlered harvest inputs to 0 and None to negate any alterations to the model. This option is best for processing large numbers of simulations because it negates the need to select each management response individually.

The *nlrx* package was developed with options for implementing NetLogo models using R code on an HPC cluster [35], and our implementation of the *nlrx* package followed Salecker [40]’s vignette with appropriate modifications. We used the Roar Collab HPC cluster managed by the Institute for Computational and Data Sciences at The Pennsylvania State University, University Park, Pennsylvania, USA. We defined the locations of our R package library, Java, NetLogo, and *PAOvCWD* similarly to the R markdown version of Step 4. We also defined our *nlrx*-formatted simulation matrix as outlined above. We then used the *clustermq* package [41] to submit jobs to the HPC cluster from within the submitted R script. This approach required building a function to define parameters for each HPC cluster worker, but model steps were otherwise similar to those implemented in the R markdown version of Step 4.

### *PAOvPOP* and *PAOvCWD*

The primary purpose of *MOOvPOP* [9,10] *MIOvPOP* ([[8], [12]]), and *INOvPOP* [22] was to initialize landscapes and corresponding populations of deer. Differences between *MOOvPOP, MIOvPOP*, and *INOvPOP* were minimal. Each model contained different landscapes that users could import, and *INOvPOP* included changes to programming language corresponding to NetLogo software updates. Otherwise, model inputs and functions were largely the same. *PAOvPOP* similarly includes updated relevant landscapes for users to choose from in addition to these more consequential modifications:

1. We annotated significant sections of the code, moved sections of code to frontload numeric inputs, and renamed some variables to make future modification easier. Annotations included adding the keyword “activate” to sections of the code that can be toggled to produce different outputs.
2. We hid a greater proportion of agents from the landscape to increase model speed when using the NetLogo interface.
3. We added several sliders to make modification of inputs easier (Fig. 2) including proportions of adult males, adult females, yearling males, and yearling females, juvenile female pregnancy rate, adult female pregnancy rate, juvenile male dispersal rate, juvenile female dispersal rate, and simulation duration.

**Fig. 2:**
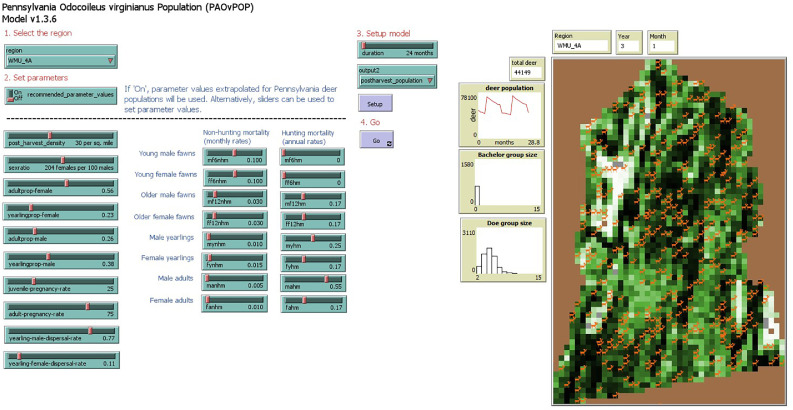
*PAOvPOP* user interface. Users are asked to select a region of interest, parameter values, simulation duration, and simulation output. Landscape visualization represents percent forest cover (Fig. 1) with most agents hidden to improve model processing speeds.
4. We altered recommended parameter values for deer movement to match literature specifically pertaining to deer populations in Pennsylvania (Table 3).

**Table 3:** Demographic descriptors of white-tailed deer (*Odocoileus virginianus*) used in *PAOvPOP* and *PAOvCWD*. Parameter names are program inputs accompanied by written descriptions. *PAOvCWD* values are those used in our models accompanied by citations supporting our decision. Parameters citing Table 1 were specific to each wildlife management unit (ranges) or the statewide average (single values) derived from the Pennsylvania sex-age-kill models [33,34].

Parameter	Description	*INOvCWD*	*PAOvCWD*	Citation
post_harvest_density	Post-harvest (January) density of deer per square mile	14	16–45	Table 1
sexratio	Number of yearling and adult females per 100 yearling and adult males	120	167–233	Table 1
adultprop-female	Proportion of post-harvest female population > 30 months of age	0.4	0.51–0.65	Table 1
adultprop-male	Proportion of post-harvest male population > 30 months of age	NA	0.17–0.39	Table 1
yearlingprop-female	Proportion of post-harvest female population 18 months of age	0.25	0.18–0.25	Table 1
yearlingprop-male	Proportion of post-harvest male population 18 months of age	NA	0.31–0.44	Table 1
mf6nhm	Monthly non-hunting male fawn mortality (0–6 months)	0.055	0.1	[[43], [44], [45]]
ff6nhm	Monthly non-hunting female fawn mortality (0–6 months)	0.055	0.1	[[43], [44], [45]]
mf12nhm	Monthly non-hunting male juvenile mortality (7–12 months)	0.05	0.03	[46]
ff12nhm	Monthly non-hunting female juvenile mortality (7–12 months)	0.05	0.03	[46]
mynhm	Monthly non-hunting male yearling mortality (12–24 months)	0.01	0.01	[43,47,48]
fynhm	Monthly non-hunting female yearling mortality (12–24 months)	0	0.015	[43,47]
manhm	Monthly non-hunting male adult mortality (> 24 months)	0.01	0.005	[43,48]
fanhm	Monthly non-hunting female adult mortality (> 24 months)	0.02	0.01	[43]
mf12hm	Annual harvest rate of male juveniles (7–12 months)	0.05	0.17	Table 1
ff12hm	Annual harvest rate of female juveniles (7–12 months)	0.02	0.17	Table 1
myhm	Annual harvest rate of male yearlings (12–24 months)	0.25	0.25	Table 1
fyhm	Annual harvest rate of female yearlings (12–24 months)	0.15	0.17	Table 1
mahm	Annual harvest rate of male adults (> 24 months)	0.4	0.55	Table 1
fahm	Annual harvest rate of female adults (> 24 months)	0.2	0.17	Table 1
juvenile-pregnancy-rate	Number of fawns produced by 100 yearling (12 months old) females	20	25	[49]
adult-pregnancy-rate	Half the number of fawns produced by 100 adult (≥ 24 months old) females	80	75	[49]
yearling-male-dispersal-rate	Proportion of yearling males that disperse	0.46	0.77	[42]
yearling-female-dispersal-rate	Proportion of yearling females that disperse	0.22	0.11	[50,51]
mean-female-dispersal-distance	Distance (miles) female deer disperse if dispersing	11	8	[50,51]
stddev-female-dispersal-distance	Standard deviation of female deer dispersal distances	4	3	[50,51]
mean-male-dispersal-distance	Distance (miles) male deer disperse if dispersing	0–21.7	0–21.7	[52]
stddev-male-dispersal-distance	Standard deviation of male deer dispersal distances	8	8	[52]
5. We modified the process of establishing the landscape and provided more detailed instructions for importing novel landscapes. Specifically, we added tools from the *csv* extension and provided an R markdown file (Step 1) that automates the process for generating the necessary rasters.
6. We broadened patch suitability so that all patches (i.e., cells) with ≥ 5 % forest cover were considered suitable deer habitat. This increased deer population estimates in the model to match real-world estimates.
7. We altered the age threshold at which density-dependent mortality equations were applied such that density-dependent mortality was implemented for deer beginning at 10 years of age to increase realism.
8. We increased carrying capacity to 100 deer per 1 mile^2^ (2.59 km^2^) to prevent model failure above carrying capacity and to increase realism of pre-harvest densities.
9. We altered mean bachelor group sizes and pregnancy rates to match those used in *INOvCWD* [14]. Previous iterations used different values for the two ABMs.
10. We altered male dispersal dynamics such that males have a greater likelihood of dispersing in May but can alternatively disperse in October if they did not disperse in May [42].

**Fig. 3:**
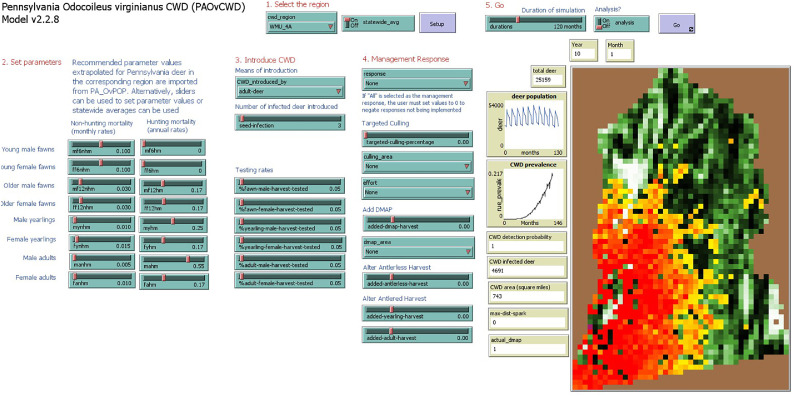
*PAOvCWD* user interface. Users are asked to select a region of interest, mortality parameter values, chronic wasting disease (CWD) introduction and monitoring dynamics, CWD management response, and simulation duration. Landscape visualization represents percent forest cover (Fig. 1) with CWD progression overlaid as the intensity of CWD as a yellow to red gradient corresponding to proportion of deer infected with CWD.

Much of the model structure underlying *MOOvPOPsurveillance* [9,10], *MIOvCWDdy* [8,12], *INOvCWD* [14], and our *PAOvCWD* model is the same as the *MOOvPOP, MIOvPOP, INOvPOP*, and *PAOvPOP* models that initialize their landscapes and agents. These similarities are necessary because variables and processes must be maintained when transferring the initialized *PAOvPOP* model to the more interactive *PAOvCWD* model. *MOOvPOPsurveillance* was originally designed to simulate natural CWD transmission and identify the monitoring thresholds needed to accurately identify areas of risk [9,11], and the *MIOvCWDdy* derivation [8,12] was minimally different. *INOvCWD* included modifications to the code corresponding to NetLogo software updates and additionally offered an option to implement targeted culling of deer in the model [14]. Our *PAOvCWD* model substantially modified *INOvCWD*; in addition to the changes listed above (excepting numbers 2 and 8 as inapplicable), we made the following modifications to the code and interface (Fig. 3):

11. Using the “activate” keyword, we added an option allowing models to be stopped upon detection of CWD.
12. We added an option where patch color is set to a scaled continuous color of yellow to red corresponding to the intensity of CWD in each cell using the *palette* extension as controlled by the analysis toggle (analysis must be turned off to use this feature as it negates some exported metrics).
13. We made it possible for male deer ≥ 18 months of age in January to be considered bachelor group leaders. This change was necessary because simulations inclusive of intensive male harvest resulted in an insufficient number of males reaching 30 months of age and qualifying as group leaders. Removing this effect also more accurately reflected male social group dynamics [53].
14. We modified the CWD progression dynamics such that deer became infectious earlier after initial exposure (3–6 months) [54,55], the clinical period was longer (3–5 months) [56], and half of fawns born to infected mothers were born with CWD [57].
15. We altered mating dynamics such that the maximum number of mating partners was increased (2–4 partners among females and immature males and 4–6 partners among mature males) [58,59], the mating search radius more accurately matched that of Pennsylvania deer, and the CWD transmission risk factor during mating was increased (10 for females and 25 for males) as mating events are high risk for CWD transmission [60] and not all mating interactions include copulation.
16. We altered monthly demographic-specific contact rates to match those observed in Pennsylvania [53].
17. We added six potential management responses and appropriate user inputs for each (Table 2; Fig. 3).

### Purpose and patterns

Descriptions of ABMs generally follow the overview, design concepts, and details (ODD) protocol [61,62], as demonstrated by prior ABMs of CWD transmission among deer [11,18,63]. In this and following sections, we describe our *PAOvPOP* and *PAOvCWD* models following ODD formatting.

The purpose of our models is to provide a framework for assessing the effectiveness of management responses for limiting the spread of CWD among deer. Our models address this goal by establishing real-world landscapes for the entire state of Pennsylvania defined by percent forest cover and populating the landscapes with a realistic approximation of the deer population present in these regions. Programmed patterns of monthly behavioral activities correspond to CWD transmission with management responses programmed to imitate potential responses to the arrival of CWD. Specific management actions include monitoring harvested deer for CWD, culling infected populations, establishing DMAP units, and altering harvest regimes.

### Entities, state variables, and scales

The regional landscapes in our model are composed of 1 mile^2^ (2.59 km^2^) cells (i.e., patches) representing each of Pennsylvania’s 22 WMUs (Fig. 1). Each patch is defined by its percent forest cover derived from the NLCD [31]. Percent forest cover is integral to the model as patches are only considered available habitat if percent forest cover is ≥ 5 %. Further, juvenile male dispersal is influenced by the percent forest cover of the patch from which the individual disperses.

These regional landscapes are populated by deer. Each deer has seven state variables including sex (male or female), age (in months), CWD status (infected, uninfected), CWD progression (months since infected), their mother’s identifier, group identifier, and social group leader status. Initial deer populations are established using estimated demographic parameters for each region (Table 1) estimated using sex-age-kill models [[33], [34]]. State variables are then used to select appropriate behavioral parameters and mortality rates (Table 3) as well as contact rates. Social group dynamics follow the establishment and maintenance of matriarchal family groups and male bachelor groups corresponding to deer behavior.

Each time step in our model represents one month, with years progressing when month advances from December to January. Each month’s simulated processes correspond to behaviors observed during these periods (e.g., mating in November and parturition in May).

### Process overview and scheduling

Processes incorporated into our model include those associated with deer biology (e.g., mating, parturition, dispersal, and social group formation) and those associated with deer management (e.g., hunter harvest, CWD monitoring, and culling) using Pennsylvania-specific values (Table 1; Table 3). These processes use the same values for both infected and uninfected deer [[53], [56], [64]] and occur on a monthly schedule. As each month (i.e., each time step) advances, deer contact other deer in their vicinity potentially transmitting CWD; deer age is increased by one month; CWD progression among infected deer increases by one month; deer experience non-hunting mortality; and, female social groups realign in response to mortalities. Months with specific processes include January, May, June, November, and December. In January, male social groups form following male isolation during the previous November and December. In May, yearling males and females disperse following appropriate dispersal rates and dynamics; yearling and adult females give birth following corresponding pregnancy rates; and, female social group dynamics are adapted corresponding to modified group sizes resultant from dispersal and parturition. In June of only the first year of the simulation, a random subsample of deer are infected with CWD following user inputs. In November, yearling males that have not yet dispersed do so, and all male social groups are disbanded while mating occurs mating dynamics are limited to their role in CWD transmission and not reflective of individual deer becoming pregnant. In December, deer are harvested corresponding to user-defined harvest rates; a random subsample of harvested deer are tested for CWD corresponding to user-defined monitoring rates; and, deer are removed via targeted culling if CWD has been detected and the user has defined parameters for culling as a management response. Once CWD has been detected, the harvest rates will change following the user-input management response.

### Design concepts

*Basic Principles*: Deer social behaviors are repeated seasonally on an annual cycle and describe potential mechanisms of CWD transmission. Management practices (e.g., hunter harvest and targeted culling) alter deer demographics and by extension deer social behavior. By altering management strategies in response to CWD detection, CWD transmission may be slowed.

*Emergence*: The prevalence of CWD in the model landscape is dependent on deer social dynamics and intraspecific transmission. Deer management practices alter these dynamics resulting in corresponding estimates of CWD prevalence.

*Adaptation*: Deer in our model adapt by selecting whether and how far to disperse and via social group dynamics corresponding to season and familial associations. Management actions influence deer social group dynamics altering these adaptations.

*Objectives, Learning*, and *Prediction*: Deer in our model do not adapt via an objective-seeking mechanism, learn from their prior actions, or predict future conditions or consequences. Management responses are similarly pre-selected and do not adapt to meet specific objectives via learning or prediction.

*Sensing*: Deer can sense the percent forest cover of their current patch as well as the presence and social organization of other deer. Specifically, deer use percent forest cover to determine patch suitability, and juvenile males use percent forest cover to determine dispersal distance. Social groups are formed among deer inhabiting the same patch and delineated by sex, age, and an individual’s mother. Deer may therefore be considered to accurately sense the sex, age, proximity, and familial relationship of other deer.

*Interaction*: Deer exhibit direct interactions with one another. The sole function of these interactions is the transmission of CWD from infected to uninfected individuals. Deer are otherwise unaffected by direct interactions, though social groups are rearranged corresponding to external stimuli. The number of interactions corresponds to social-demographic contact rates mediated by social group status and season. In brief, deer more frequently contact members of their social group than deer outside their social group. Within matriarchal (i.e., antlerless) social groups, deer contact their mother and siblings more frequently than unrelated individuals. Interactions during mating occur between males and females not member to the same social group.

*Stochasticity*: Stochasticity is incorporated into several processes. Monthly non-harvest mortality and annual hunter harvest mortality (as well as targeted culling mortality, if implemented) are stochastic processes. Some social group dynamics are mediated by stochastic processes including the probability, distance, and direction of dispersal, social group size, group selection by dispersing individuals, group restructuring following mortalities, and parturition. Mating incorporates stochasticity via the number of mating partners. The rate of CWD progression and the probability of transmitting CWD from an infected to an uninfected individual are also moderated by stochastic processes.

*Collectives*: Social groups (i.e., matriarchal antlerless and bachelor antlered social groups) are integral to the transmission of CWD. These social groups influence intraspecific contact rates. Social group membership is influenced by numerous aspects of our model structure (e.g., sex, age, dispersal, and mortality).

*Observation*: The primary purpose of our model is to assess the prevalence of CWD in response to management actions. CWD prevalence may be observed via the proportion of deer infected with CWD (i.e., number of infected deer / total number of deer), the number of patches in which a deer is positive for CWD (i.e., area of infection in miles^2^), and the distance from the initially infected patch to the furthest infected patch (miles). The total number of deer is also observed—total population is useful for assessing the influence of management responses on deer density (i.e., total deer / total suitable patches).

### Initialization

The *PAOvPOP* model is used to initialize the population. The landscape is initialized by importing a prebuilt ascii file defining the patches. Patches are valued corresponding to percent forest cover. Deer are established on this landscape where patches with < 5 % forest cover, an immutable value in our models, are considered unsuitable habitat and cannot be populated. The total number of deer generated corresponds to the user-input for deer density (deer / miles^2^) multiplied by the number of available patches. Proportions of deer of various sex and age classes are similarly estimated via user inputs. If the recommended parameter values are used, values are specific to the WMU selected for the simulation (Table 1). Suitable patches are systematically filled to the assigned density via a stochastic process of age and sex class selections based on the demographic proportions. Initialization occurs at the start of a new year (i.e., before January processes are implemented). Therefore, all deer are initially 6, 18, or ≥ 30 months of age. Initial doe social groups are formed as part of the initialization process, while male social groups are formed during the January time step processes. Following simulation of population dynamics using *PAOvPOP, PAOvCWD* imports the final *PAOvPOP* landscape. No additional initialization steps occur as part of the *PAOvCWD* setup. It is therefore necessary for *PAOvPOP* simulations to stop at the end of December prior to January model processes.

### Input data

The model does not use input data to represent time-varying processes.

### Submodels

*CWD Introduction*: In June of the first year, CWD is introduced to the existing deer population and users input the number of deer and the demographic class (adult deer, dispersing yearling males, dispersing yearling females, females in a social group, females without a social group, males in a social group, or males without a social group) that will be infected. A patch considered suitable deer habitat is randomly selected and the user-selected number of deer in the corresponding patch and user-selected demographic class are infected with CWD. If the user selects a number greater than the total number of deer in the selected patch and demographic class, all qualified deer within that patch will be infected. The CWD progression counter for these initially-selected deer is set to 5–10 months.

*CWD Progression*: When a deer becomes infected with CWD, it is assigned intrinsic values for the potential durations of their pre-infectious phase (i.e., months from exposure to becoming infectious; 2–6 months [54,55]), their pre-clinical phase (i.e., months from becoming infectious to presenting clinical signs of CWD; 20–25 months [65]), and their survival (i.e., months from exposure to mortality due to CWD; 2–4 months + duration of the pre-clinical phase [56]). If a deer experiences non-CWD mortality prior to reaching the duration limit of CWD mortality, CWD mortality is voided. Excluding initially selected CWD-infected deer, other deer are infected via intraspecific contact. When a deer becomes infected, their CWD progression counter is set to 0. When each month progresses, the CWD progression counter of infected deer is increased by 1 month.

*Contact Rates*: Deer experience intraspecific contact each month following estimates of contact rates among deer in Pennsylvania with corrections corresponding to *INOvCWD* model inputs [[14], [53]]. The probability of transmitting CWD during each contact event is 1.28 % [[9], [10], [14], [66]].

*Male Social Groups*: Male bachelor groups are composed of ≤ 4 males within 1.5 miles of one another. Male bachelor groups occur only during January–October and are disbanded during November–December. During the intact January–October period, the only alterations to male group dynamics occur if a male group member dies due to non-hunting mortality, a dispersing male joins a group, or multiple dispersing males form a new group.

*Female Social Groups*: Matriarchal female group size is limited to 6 deer at the time of parturition; this maximum typically corresponds to an eldest female, her daughter(s) from the previous year, her offspring from the current year, and her daughter(s)’s offspring from the current year. If this total number exceeds 6 due to parturition, the yearling daughter(s) typically form new social groups distinct from their mother’s existing social group.

*Male Dispersal*: We modeled male dispersal such that approximately 32 % of yearling males disperse during May, 45 % disperse during November, and 23 % of males remain in their natal range but are no longer members of their natal social groups [42]. Male dispersal distance is determined by the percent forest cover of the patch from which they are dispersing. Dispersal distance follows the equation: distance = 35.07 (± 2.05) - 48.14 (± 4.34) * percent forest cover [52]. Males that disperse outside the defined WMU habitat patches are replaced by dispersing males entering the population with the same intrinsic characteristics but new individual and group identifiers.

*Female Dispersal*: Our model is set up so that 11 % of yearling females disperse from their natal range in May. These dispersing females move 5–11 miles from their natal range [50,51]. Females that disperse outside the defined WMU habitat patches are replaced by dispersing females entering the population with the same intrinsic characteristics but new individual and group identifiers. Females that do not disperse remain members of their natal social groups until dynamics governing matriarchal social groups alter this membership.

*Parturition*: In May, 75 % of adult females give birth to two offspring, and 25 % of yearling females give birth to a single offspring [49]. The sex of all offspring is evenly split between male and female. If an offspring is born to a mother infected with CWD, the offspring has a 50 % chance of being born infected with CWD [57]. Our model does not monitor nor require females to have mating contact in the prior year to give birth to offspring; we ignored this biological necessity because our reproductive rates (i.e., 75 % and 25 %) account for not all females becoming pregnant. Offspring immediately become members of their mothers’ social groups, with matriarchal social group dynamics adjusting in response.

*Mating*: At the onset of mating, adult males, yearling males, and females are assigned a number (4–6, 2–4, and 2–4, respectively) corresponding to the number of opposite sex partners with whom they are willing to mate [58,59]. Males are also assigned a search radius value (1–2 miles) within which they can contact females. Males and females are then systematically paired until there are no available opposite sex partners within each deer’s search radius who are still willing to mate with more members of the opposite sex. For each of these pairs, the probability of transmitting CWD from an infected male to an uninfected female is 12.8 %, and the probability of transmitting CWD from an infected female to an uninfected male is 32.0 %.

*Non-Hunting Mortality*: As each month progresses, deer experience mortality based on their sex and age classes (Table 3). The mechanism for determining monthly survival is a stochastic process wherein each individual must pass a mortality check corresponding to these monthly percentages. Following a mortality, social group dynamics are adapted following appropriate mechanisms outlined above. If a female with offspring ≤ 2 months of age dies, her offspring also die.

*Hunter Harvest*: Hunter harvest occurs during December in our model. The mechanism for determining harvest is a stochastic process wherein each individual must pass a mortality check corresponding to these monthly percentages. Following mortalities, social group dynamics are modified following the rules described above.

*Monitoring*: Monitoring rates are demographic-specific and input by users. Monitoring occurs as part of the hunter harvest process. Each deer harvested maintains its age and sex classes as they correspond to the monitoring rates for each respective demographic designation. Each harvested deer then passes a monitoring check as part of a stochastic process. If a deer is infected with CWD and is selected to be tested via the monitoring process, the CWD detection parameter is altered from 0 to 1 indicating CWD has been detected in the WMU; additionally, the location of the deer that tested positive is recorded. In the following year(s) a management response is implemented.

*Management Response*: Once CWD has been detected, a user-defined management response is implemented: no response, targeted culling, adding a DMAP subunit, altering antlerless harvest, altering antlered harvest, and all (Table 2).

## Method validation

Most of the input parameters used to define our model were based on research conducted on free-ranging deer in Pennsylvania (Table 3). The PGC initially detected CWD among three free-ranging deer in WMU 4A in 2012 via a statewide CWD monitoring initiative [67]. The PGC attempted to implement a management response by creating DMAP 2874 and performing targeted culling [38], but their effect on the deer population was negligible due to extenuating circumstances. Since 2012, CWD has been monitored in WMU 4A [39], and because management responses were restricted, WMU 4A served as a case study to validate our model. The code used for model validations was published as Step 5 in Wehr et al. [23].

**Fig. 4:**
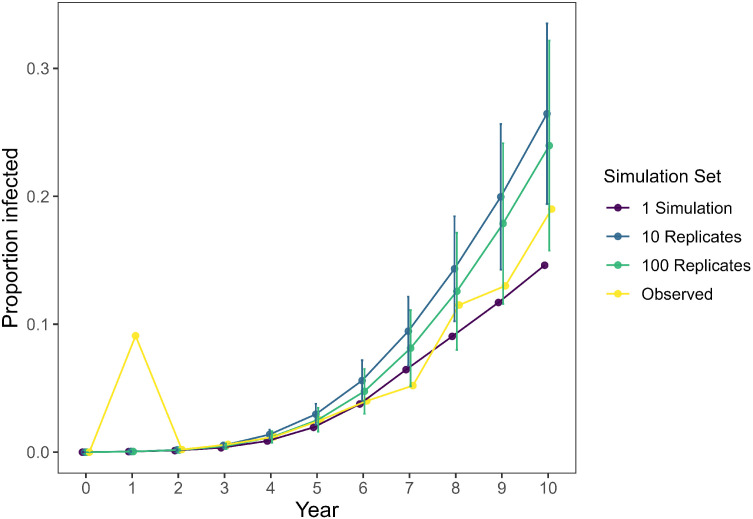
Estimates of chronic wasting disease (CWD) prevalence in wildlife management unit 4A of Pennsylvania, USA. Observed estimates (yellow) of the proportion of white-tailed deer (*Odocoileus virginianus*) infected with CWD were derived from monitoring efforts led by the Pennsylvania Game Commission [39]. Simulated estimates were developed using *PAOvCWD* with varying levels of replication (1, 10, or 100 replications) with error bars representing 95 % confidence intervals. The high proportion infected estimated in year 1 corresponds to a low sampling effort that year [39].

**Fig. 5:**
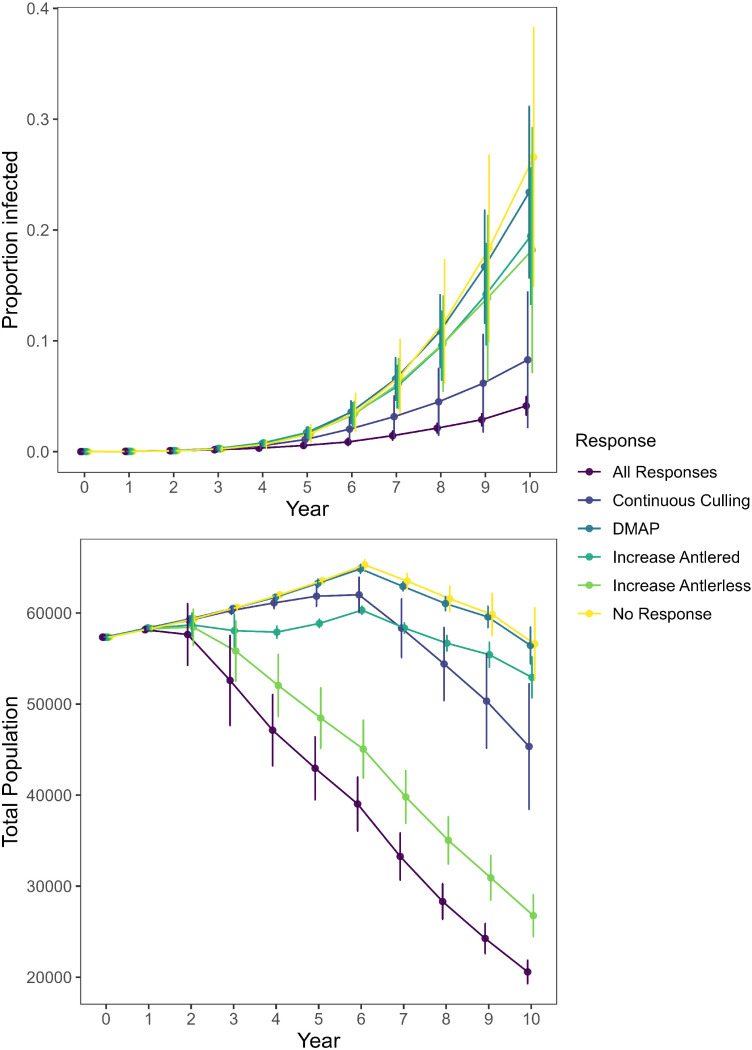
Estimates of chronic wasting disease prevalence and total number of white-tailed deer (*Odocoileus virginianus*) in wildlife management unit (WMU) 1B, Pennsylvania, USA. Estimates and 95 % confidence intervals were calculated for management responses including: no response, increased antlerless harvest (8 % increase for entire WMU), increased antlered harvest (25 % increase in yearling male and 10 % increased in adult male harvest for entire WMU), increased antlerless harvest within a deer management assistance program (DMAP) subunit (10 % increase in 49 mile^2^ (126.91 km^2^) area), continuous culling (25 % of post-harvest deer in 1 mile^2^ (2.59 km^2^) area each year), and all management responses simultaneously.

**Fig. 6:**
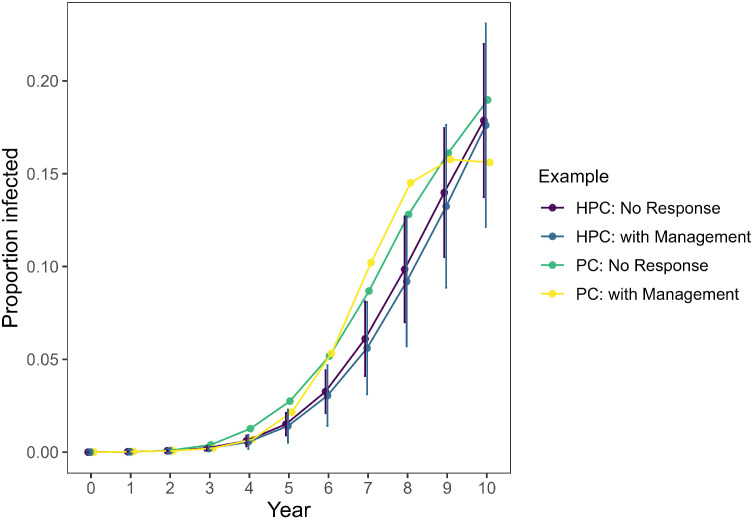
Estimates of chronic wasting disease prevalence among white-tailed deer (*Odocoileus virginianus*) in wildlife management unit (WMU) 3A, Pennsylvania, USA. Estimates and 95 % confidence intervals were produced using *PAOvCWD* on a personal computer (PC) or high-performance computing (HPC) cluster. Simulated scenarios included those with no management response and those with a management response inclusive of an initial cull (50 % of post-harvest deer within 1 mile^2^ of the detection location) and increased antlerless harvest (3 % increase for entire WMU).

Following an initially high estimate of the prevalence of CWD in WMU 4A due to the small sample size, estimated CWD prevalence in WMU 4A rose steadily during the first 10 years following initial detection [39]. Our model simulation closely matched this trend with observed estimates falling within the lower bounds of the confidence intervals generated by our simulations (Fig. 4). Given that our model coincided with observed values without the need to modify any of the model input values obtained from the literature (Table 3), we elected not to modify our model to bring our model’s estimates closer to observed values. In addition to validating our model using WMU 4A, we confirmed that our model produced plausible results for all of the potential management responses (Fig. 5). Further, we confirmed our model will produce similar outputs using either a personal computer or HPC cluster (Fig. 6).

## Limitations

Careful consideration of user applications and limitations is warranted when applying ABMs [61,62]. We constructed our model such that as many defining aspects as possible were derived from research conducted in Pennsylvania (Table 3). This approach likely strengthens our model in comparison to previous iterations in which values were derived from geographically disparate sources or via expert opinion. However, this may limit effective implementations of our model to temperate regions of the eastern United States because our model may be ineffective in other regions where deer ecology is different (e.g., the search radius of males during rut is greater in Texas than in Pennsylvania [68]).

Annual population growth rate in our model is sensitive to female survival rates. Using statewide averages (Table 1, Table 3) results in populations with a slightly positive growth rate that is counteracted by CWD-mediated mortality beginning around year 5 in most simulations (Fig. 5). However, WMUs with female survival rates on the high or low end of the range of available values have annual population growth rates that are correspondingly higher or lower than the statewide average. Because these starting values for mortality are generally maintained for the duration of a simulation, the application of WMU-specific mortality rates is more variable than using statewide averages.

Our model limits CWD transmission to direct contact events and does not consider indirect contact. Though indirect contact via natural and anthropogenic surfaces has been identified as a potential mechanism of CWD transmission [69,70], estimation of indirect contact in the context of ABMs [17,18] and movement ecology [71,72] is relatively limited [53]. Indirect contact is likely a lesser pathway for CWD transmission in early stages prior to the development of a sufficient environmental reservoir of CWD prions [[17], [73]] and among deer populations with densities similar or less than those typically observed in Pennsylvania [74]. As such, our model should likely be limited to relatively short-term simulations (i.e., approximately 10 years). Further, when we attempted simulations of longer duration, we observed a point at which CWD prevalence was high enough that CWD-mediated mortalities began occurring more quickly than the population could reproduce resulting in the death of all deer in our models, which did not occur until after our suggested 10-year threshold. Though this effect on population growth rate is supported by existing literature from Wyoming, USA [75], it is unlikely populations will be entirely extirpated as CWD has been present among deer populations for many decades [2]. This reality may, in part, be due to the presence of a CWD-resistant gene present in some deer that results in longer lifespans among infected individuals [[76], [77]], a factor not considered in our model.

A final limitation of our model is the significant processing time necessary to complete simulations at the WMU-scale. Our model simulates the entire deer population, and WMUs, particularly those with high deer densities, often have > 50,000–100,000 agents (Table 1). As such, running simulations often required hours on a personal computer. We overcame this challenge by using an HPC cluster to improve processing speeds and parallelize many simulations with a relatively low failure rate—the HPC cluster failed to process a simulation without reporting a distinct error in 8 of 1,011 (0.8 %) validation simulations. For users without HPC cluster access, simulations using smaller study areas and therefore smaller agent sets may be the prefered option. To facilitate this user experience, a NetLogo-formatted raster of percent forest cover representing DMAP 2874 (375 miles^2^; 971.25 km^2^) was included in the software release [23]. This smaller study area allows users to run simulations within the NetLogo user interface more quickly and improves the ability of users to develop an interactive understanding of how *PAOvCWD* works.

## CRediT authorship contribution statement

**Nathaniel H. Wehr:** Conceptualization, Methodology, Software, Validation, Data curation, Writing – original draft, Writing – review & editing, Visualization. **Christopher S. Rosenberry:** Conceptualization, Writing – review & editing, Funding acquisition. **David Stainbrook:** Conceptualization, Data curation, Writing – review & editing. **Maureen Staats:** Conceptualization, Writing – review & editing. **Andrea L. Korman:** Conceptualization, Writing – review & editing. **W. David Walter:** Conceptualization, Methodology, Data curation, Writing – review & editing, Funding acquisition, Resources.

## Declaration of competing interest

The authors declare that they have no known competing financial interests or personal relationships that could have appeared to influence the work reported in this paper.

## Data Availability

Data associated with this method was published by Wehr et al. 2025 [23].

## References

[1] Pritzkow S. (2022). Transmission, strain diversity, and zoonotic potential of chronic wasting disease. Viruses..

[2] Richards B.J. (2021). Chronic Wasting Disease Distribution in the United States By State and County (ver. 3.0, June 2025).

[3] Thompson N.E., Huang M.H.J., Christensen S.A., Demarais S. (2023). Wildlife agency responses to chronic wasting disease in free-ranging cervids. Wildl. Soc. Bull..

[4] Thompson N.E., Mason J.R. (2022). The cost of combatting chronic wasting disease. Wildl. Prof..

[5] Conover M.R., Hewitt D.G. (2011). Biology and Management of White-Tailed Deer.

[6] Hewitt D.G. (2015). Hunters and the conservation and management of white-tailed deer (*Odocoileus virginianus*). Int. J. Environ. Stud..

[7] Rivera N.A., Brandt A.L., Novakofski J.E., Mateus-Pinilla N.E. (2019). Chronic wasting disease in cervids: prevalence, impact and management strategies. Vet. Med.: Res. Rep..

[8] Belsare A. (2019).

[9] Belsare A., Gompper M., Keller B., Sumners J., Hansen L., Millspaugh J. (2020). Size matters: sample size assessments for chronic wasting disease surveillance using an agent-based modeling framework. MethodsX..

[10] Belsare A., Gompper M., Millspaugh J.J. (2020).

[11] Belsare A.V., Gompper M.E., Keller B., Sumners J., Hansen L., Millspaugh J.J. (2020). An agent-based framework for improving wildlife disease surveillance: a case study of chronic wasting disease in Missouri white-tailed deer. Ecol. Modell..

[12] Belsare A.V., Stewart C.M. (2020). OvCWD: an agent-based modeling framework for informing chronic wasting disease management in white-tailed deer populations. Ecol. Solut. Evid..

[13] Wilensky U., NetLogo (1999). Center for Connected Learning and Computer-Based Modeling at Northwestern.

[14] Belsare A. (2024).

[15] Strasburg M., Christensen S. (2024). Evaluating the interaction of emerging diseases on white-tailed deer populations using an agent-based modeling approach. Pathogens..

[16] Mysterud A., Viljugrein H., Rolandsen C.M., Belsare A.V. (2021). Harvest strategies for the elimination of low prevalence wildlife diseases. R. Soc. Open. Sci..

[17] Kjær L.J., Schauber E.M. (2022). The effect of landscape, transmission mode and social behavior on disease transmission: simulating the transmission of chronic wasting disease in white-tailed deer (*Odocoileus virginianus*) populations using a spatially explicit agent-based model. Ecol. Modell..

[18] Thompson N.E., Butts D.J., Murillo M.S., O'Brien D.J., Christensen S.A., Porter W.F., Roloff G.J. (2024). An individual-based model for direct and indirect transmission of chronic wasting disease in free-ranging white-tailed deer. Ecol. Modell..

[19] Gritter K., Dobbin M., Merrill E., Lewis M. (2024). An individual-based movement model for contacts between mule deer (*Odocoileus hemionus*). Ecol. Complex..

[20] North M.J., Collier N.T., Ozik J., Tatara E.R., Macal C.M., Bragen M., Sydelko P. (2013). Complex adaptive systems modeling with Repast Simphony. Complex Adapt. Syst. Model..

[21] R Core Team (2025). R: a Language and Environment for Statistical Computing.

[22] Belsare A. (2024). INOvPOP (version 1.1.1).

[23] Wehr N.H., Rosenberry C.S., Stainbrook D., Staats M., Korman A.L. (2025). Software Release.

[24] Wickham H., Fracois R., Henry L., Muller K., Vaughan D. (2023).

[25] Bocinsky R.K. (2025).

[26] Pebesma E. (2018). Simple features for R: standardized support for spatial vector data. R J..

[27] Pebesma E., Bivand R. (2023).

[28] Wickham H., Hester J., Bryan J. (2024).

[29] Hijmans R. (2024).

[30] PGC, Wildlife Management Units. Pennsylvania Game Commission, Harrisburg, PA, 2021 Accessed 17 July 2025 https://www.pasda.psu.edu/uci/DataSummary.aspx?dataset=1122.

[31] J. Dewitz, USGS, National Land Cover Database (NLCD) 2019 Products, United States Geological Survey, Reston, Virginia, USA, 2021, doi:10.5066/P9JZ7AO3.

[32] Evans T.S., Kirchgessner M.S., Eyler B., Ryan C.W., Walter W.D. (2016). Habitat influences distribution of chronic wasting disease in white-tailed deer. J. Wildl. Manag..

[33] Rosenberry C.S., Fleegle J.T., Wallingford B.D. (2011). Monitoring Deer Populations in Pennsylvania.

[34] Stainbrook D., Wallingford B., Fleegle J.T., Weiss P., Lupo P. (2024). Deer health, Forest Habitat health, Deer harvests, and Deer Population Trends By Wildlife Management Unit.

[35] Salecker J., Sciaini M., Meyer K.M., Wiegand K. (2019). The nlrx r package: a next-generation framework for reproducible NetLogo model analyses. Methods Ecol. Evol..

[36] Salecker J. (2025). https://docs.ropensci.org/nlrx/articles/getstarted.html.

[37] Bengtsson H. (2021). A unifying framework for parallel and distributed processing in R using futures. R. J..

[38] PGC (2020). Chronic Wasting Disease Response Plan.

[39] PGC (2025). https://pagame.maps.arcgis.com/apps/dashboards/b3c0fd44cc5944ebbc2229ede897b2ae.

[40] Salecker J. (2025). https://docs.ropensci.org/nlrx/articles/furthernotes.html.

[41] Schubert M. (2019). clustermq enables efficient parallelization of genomic analyses. Bioinformatics..

[42] Long E.S., Diefenbach D.R., Rosenberry C.S., Wallingford B.D. (2008). Multiple proximate and ultimate causes of natal dispersal in white-tailed deer. Behav. Ecol..

[43] Buderman F.E. (2012).

[44] Gingery T.M. (2018). Thesis.

[45] Vreeland J.K., Diefenbach D.R., Wallingford B.D. (2004). Survival rates, mortality causes, and habitats of Pennsylvania white-tailed deer fawns. Wildl. Soc. Bull..

[46] PGC (2025). https://www.pa.gov/agencies/pgc/wildlife/discover-pa-wildlife/white-tailed-deer/predation-and-deer-population.html.

[47] Norton A.S. (2010). Thesis.

[48] Wallingford B.D., Diefenbach D.R., Long E.S., Rosenberry C.S., Alt G.L. (2017). Biological and social outcomes of antler point restriction harvest regulations for white-tailed deer. Wildl. Monogr..

[49] Diefenbach D.R., Alt G.L., Wallingford B.D., Rosenberry C.S., Long E.S. (2019). Effect of male age structure on reproduction in white-tailed deer. J. Wildl. Manag..

[50] Jennelle C.S., Walter W.D., Crawford J., Rosenberry C.S., Wallingford B.D. (2022). Movement of white-tailed deer in contrasting landscapes influences management of chronic wasting disease. J. Wildl. Manag..

[51] Lutz C.L., Diefenbach D.R., Rosenberry C.S. (2015). Population density influences dispersal in female white-tailed deer. J. Mammal..

[52] Long E.S., Diefenbach D.R., Rosenberry C.S., Wallingford B.D., Grund M.D. (2005). Forest cover influences dispersal distance of white-tailed deer. J. Mammal..

[53] Wehr N.H., Bondo K.J., Rosenberry C.S., Stainbrook D., Wallingford B.D. (2026). Intraspecific contact among white-tailed deer: a literature review and chronic wasting disease case study. Ecol. Evol..

[54] Denkers N.D., McNulty E.E., Kraft C.N., Nalls A.V., Westrich J.A., Hoover E.A., Mathiason C.K. (2024). Temporal characterization of prion shedding in secreta of white-tailed deer in longitudinal study of chronic wasting disease, United States. Emerg. Infect. Dis..

[55] Henderson D.M., Denkers N.D., Hoover C.E., Garbino N., Mathiason C.K., Hoover E.A. (2015). Longitudinal detection of prion shedding in saliva and urine by chronic wasting disease-infected deer by real-time quaking-induced conversion. J. Virol..

[56] Edmunds D.R., Albeke S.E., Grogan R.G., Lindzey F.G., Legg D.E., Cook W.E., Schumaker B.A., Kreeger T.J., Cornish T.E. (2018). Chronic wasting disease influences activity and behavior in white-tailed deer. J. Wildl. Manag..

[57] Nalls A.V., McNulty E.E., Mayfield A., Crum J.M., Keel M.K., Hoover E.A., Ruder M.G., Mathiason C.K. (2021). Detection of chronic wasting disease prions in fetal tissues of free-ranging white-tailed deer. Viruses..

[58] Buderman F.E., Gingery T.M., Diefenbach D.R., Gigliotti L.C., Begley-Miller D., McDill M.M., Wallingford B.D., Rosenberry C.S., Drohan P.J. (2021). Caution is warranted when using animal space-use and movement to infer behavioral states. Mov. Ecol..

[59] Turner M.M., Deperno C.S., Booth W., Vargo E.L., Conner M.C., Lancia R.A. (2016). The mating system of white-tailed deer under Quality Deer Management. J. Wildl. Manag..

[60] Lavelle M.J., Fischer J.W., Phillips G.E., Hildreth A.M., Campbell T.A., Hewitt D.G., Hygnstrom S.E., Vercauteren K.C. (2014). Assessing risk of disease transmission: direct implications for an indirect science. BioScience.

[61] Grimm V., Berger U., Bastiansen F., Eliassen S., Ginot V., Giske J., Goss-Custard J., Grand T., Heinz S.K., Huse G. (2006). A standard protocol for describing individual-based and agent-based models. Ecol. Modell..

[62] Grimm V., Railsback S.F., Vincenot C.E., Berger U., Gallagher C., DeAngelis D.L., Edmonds B., Ge J., Giske J., Groeneveld J. (2020). The ODD protocol for describing agent-based and other simulation models: a second update to improve clarity, replication, and structural realism. J. Artif. Soc. Soc. Simul..

[63] Van Buskirk A.N., Rosenberry C.S., Wallingford B.D., Domoto E.J., McDill M.E., Drohan P.J., Diefenbach D.R. (2021). Modeling how to achieve localized areas of reduced white-tailed deer density. Ecol. Modell..

[64] Huang M.H.J., Demarais S., Strickland B.K., Houston A., Banda A., VerCauteren K.C. (2024). Chronic wasting disease effects on a breeding season behavior in white-tailed deer (*Odocoileus virginianus*). J. Mammal..

[65] Johnson C.J., Herbst A., Duque-Velasquez C., Vanderloo J.P., Bochsler P., Chappell R., McKenzie D. (2011). Prion protein polymorphisms affect chronic wasting disease progression. PLoS One.

[66] Kjær L.J. (2010).

[67] Evans T.S., Schuler K.L., Walter W.D. (2014). Surveillance and monitoring of white-tailed deer for chronic wasting disease in the northeastern United States. J Fish. Wildl. Manag..

[68] Foley A.M., DeYoung R.W., Hewitt D.G., Hellickson M.W., Gee K.L., Wester D.B., Lockwood M.A., Miller K.V. (2015). Purposeful wanderings: mate search strategies of male white-tailed deer. J. Mammal..

[69] Huang M.H.J., Demarais S., Banda A., Strickland B.K., Welch A.G., Hearst S., Lichtenberg S., Houston A., Pepin K.M., VerCauteren K.C. (2024). Expanding CWD disease surveillance options using environmental contamination at deer signposts. Ecol. Solut. Evid..

[70] Huang M.H.J., Demarais S., Schwabenlander M.D., Strickland B.K., VerCauteren K.C., McKinley W.T., Rowden G., Valencia Tibbitts C.C., Gresch S.C., Lichtenberg S.S. (2025). Chronic wasting disease prions on deer feeders and wildlife visitation to deer feeding areas. J. Wildl. Manag..

[71] Rustand M. (2010).

[72] Schauber E.M., Storm D.J., Nielsen C.K. (2007). Effects of joint space use and group membership on contact rates among white-tailed deer. J. Wildl. Manag..

[73] Almberg E.S., Cross P.C., Johnson C.J., Heisey D.M., Richards B.J. (2011). Modeling routes of chronic wasting disease transmission: environmental prion persistence promotes deer population decline and extinction. PLoS One.

[74] McClure W.J., Powell J. (2025). Effects of transmission pathways, immunological thresholds, and long-distance dispersal on infectious spread: chronic wasting disease case study. Theor. Ecol..

[75] Edmunds D.R., Kauffman M.J., Schumaker B.A., Lindzey F.G., Cook W.E., Kreeger T.J., Grogan R.G., Cornish T.E. (2016). Chronic wasting disease drives population decline of white-tailed deer. PLoS One.

[76] Moazami-Goudarzi K., Andréoletti O., Vilotte J.L., Béringue V. (2021). Review on PRNP genetics and susceptibility to chronic wasting disease of Cervidae. Vet. Res..

[77] Ketz A.C., Robinson S.J., Johnson C.J. (2021). Pathogen-mediated selection and management implications for white-tailed deer exposed to chronic wasting disease. J. Appl. Ecol..

